# Evidence for robots

**DOI:** 10.1051/sicotj/2017020

**Published:** 2017-05-25

**Authors:** Ravikiran Shenoy, Dinesh Nathwani

**Affiliations:** 1 Imperial College Healthcare NHS Trust London W2 1NY UK

**Keywords:** Robots, Orthopaedics, Hip, Knee, Arthroplasty

## Abstract

Robots have been successfully used in commercial industry and have enabled humans to perform tasks which are repetitive, dangerous and requiring extreme force. Their role has evolved and now includes many aspects of surgery to improve safety and precision. Orthopaedic surgery is largely performed on bones which are rigid immobile structures which can easily be performed by robots with great precision. Robots have been designed for use in orthopaedic surgery including joint arthroplasty and spine surgery. Experimental studies have been published evaluating the role of robots in arthroscopy and trauma surgery. In this article, we will review the incorporation of robots in orthopaedic surgery looking into the evidence in their use.

## Introduction

With surgery increasingly using technology and instruments adapted from mechanical industry it was logical to expand the role of robots to surgery. Accordingly, robots have been designed to function autonomously or with human guidance to perform complex surgery. Although orthopaedic surgery is largely performed on bones which are rigid immobile structures, the presence of nerves, blood vessels and other soft tissue structures in the vicinity along with the natural anatomical variations necessitates in many instances the desire for human guidance and robots to help to make the surgery both accurate and safe.

## Historical aspects

The PUMA 560, a robot, was used in 1985 to place a needle for brain biopsy under computed tomography (CT) guidance [1]. This robot was used to perform transurethral resection of prostrate three years later [2]. The first laparoscopic cholecystectomy using robots was performed in 1987. The PUMA was an industrial robot designed to operate without contact with humans in a large and unconstrained environment. These pioneering procedures eventually led to the development of PROBOT at Imperial College London in 1988 specifically designed for transurethral resection of prostrate. The concept of ROBODOC was introduced in 1986 by the Sacramento veterinarian, Dr Howard “Hap” Paul, and Dr William Bargar, an orthopaedic surgeon (Integrated SurgicalSystems (ISS), Inc. of Sacramento, California, USA), to perform precision machining of the femur during hip replacement surgery [3]. This was the first surgical robot approved by the Food and Drug Administration (FDA) and in 1992 became the first robot to assist in human total hip replacement surgery.

**Figure 1.**
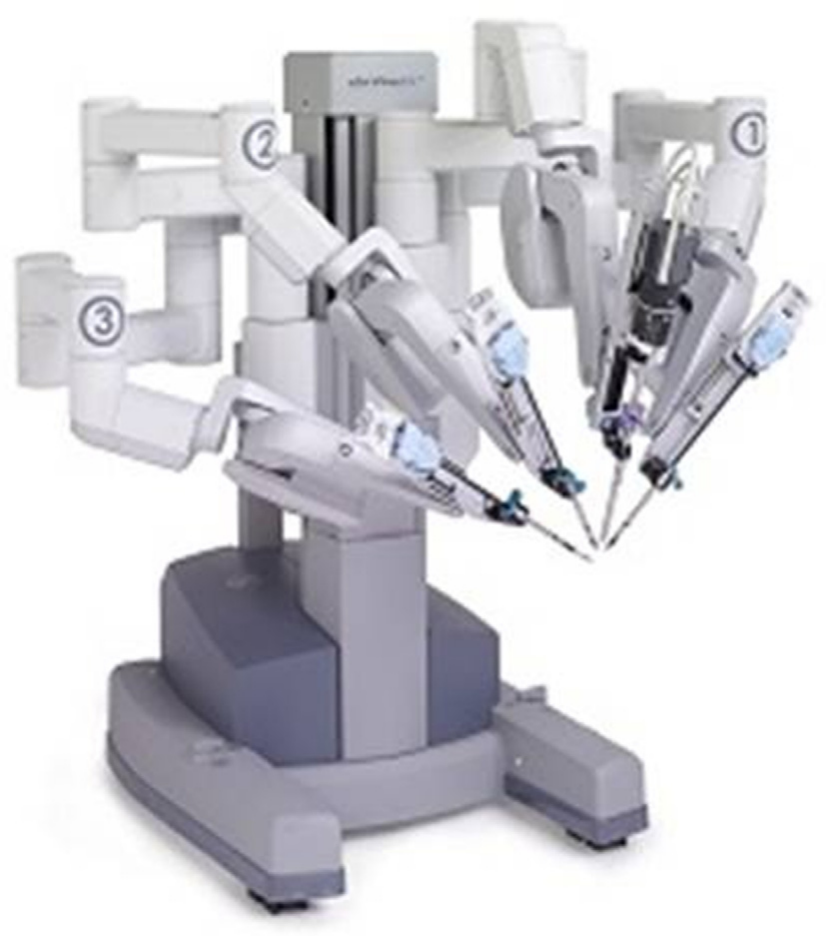
Da Vinci robot used in urological and general surgery.

Integrated Surgical Systems (now Intuitive Surgical) of Mountain View, CA, redesigned and reintroduced this technology as the Da Vinci surgical system (Figure 1). Other robots have since been developed which include ACROBOT for computer-assisted 3D planning, navigation and surgeon controlled robotic surgery.

Robots in surgery can be broadly classified autonomous or teleoperated (haptic, surgeon guided). Systems in use in orthopaedics are largely the haptic or passive robotic systems [4, 5]. Robot-assisted surgery has been studied and described in hip and knee arthroplasty, spinal surgery, foot and ankle injections, arthroscopy and trauma. The two main Robot systems currently in use are the MAKO Robot (Stryker^®^) and the NAVIO Robot (Smith & Nephew^®^) which focus currently on hip and knee procedures.

## Evidence for robots

### Hip arthroplasty

To achieve precise preparation of the fixation surface and ensure a perfect fit of the implant in a cementless total hip arthroplasty (THA) which would allow better bone ingrowth, reduce failure and thigh pain, an image-directed surgical robot for preparation of the femoral canal was described by Paul et al. [3]. Jerosch et al. in a cadaveric study where 14 femurs were randomised to have manual or robot implantation (Robodoc) of the femoral component with the aid of a software packageOrthodoc for preoperative planning showed a more accurate reproduction of the anteversion angle in the Robodoc group [6]. Börner et al. described their initial encouraging postoperative results in 300 patients who underwent robot-assisted hip endoprosthesis implantation [7]. In their prospective clinical trial with 71 patients (75 hips) divided into two groups who underwent cementless THA, the incidence of severe embolic events was lower in the Robodoc group than the conventional group [8]. Honl et al. published results of a prospective randomised study comparing robotic-assisted and manual implantation of THA in 154 patients, 80 of whom underwent manual implantation. The Robodoc system was used and the patients were followed up clinically and radiologically up to 24 months. Eighteen percent of attempted robotic implantation had to be converted to manual implantation due to failure of the system. The duration of the robotic procedures was longer than that of the manual procedures. Limb-length equality and varus-valgus orientation of the stem were better after the robotic procedures. Although the group treated with robotic implantation had a better Mayo clinical score at six and 12 months and a better Harris score at 12 months, there were no differences between the groups with any of the three scores at 24 months. Dislocation was more frequent in the group treated with robotic implantation. Recurrent dislocation and pronounced limping were indications for revision surgery in eight of the 61 patients treated with robotic implantation compared with none of the 78 with manual implantation. They recommended further development of the technology before justifying its use due to the high complication rate [9]. Schulz et al. reported on the outcome of 143 consecutive total hip replacements (128 patients) performed using the Robodoc system with a complete follow-up in 97 hips at a mean follow-up period of 3.8 years. They concluded that while the Orthodoc/Robodoc system achieves equal results as compared to a manual technique there was a high number of technical complications directly or indirectly related to the robot [10].

Most studies on robotic hip arthroplasty have been conducted on improving femoral and acetabular milling and improving fixation of a cementless stem. Domb et al. published results of their study comparing radiographic cup positioning in the safe zones described by Lewinnek et al. and Callnan et al. of robotic-assisted total hip arthroplasty (69 patients) with a matched-pair control group of conventional THAs (62 patients) performed by the same surgeon through the same posterior approach. They included 50 total hip arthroplasties in each group after exclusions. In the robotic-assisted group, 100% of cases had acetabular cups within the safe zone of Lewinnek et al. and 92% were within the safe zone of Callanan et al. Only 80% and 62% of conventional group, respectively, were within these zones. Whether this accurate placement of the acetabular cup would yield clinical benefits of reducing dislocation rates, component impingement, bearing surface wear and revision rates was unclear [11].

In summary therefore the modern use of robotic hip arthroplasty is still in the developmental stage and while the accuracy of implantation appears to be consistent there remain significant concerns around the complication rates.

### Knee arthroplasty

Martelli et al. in experimental studies on cadavers and volunteers, using computers connected to CT scanners with the surgeon using a constrained guide held by a robot, showed that the accuracy of implant can be improved with reduction in operating time and surgical errors [12]. The following year (2001) details of the first clinical application of a robotic system “ACROBOT” and results of two preliminary clinical trials demonstrating the accuracy of anatomic registration and bone cutting were published [13].

Karia et al. reported on the advantages of improved implant positioning using robot assistance by inexperienced surgeons. Sixteen surgeons were randomised to constrained robot-assisted or conventional unicompartmental knee replacement on dry bones over a period of three weeks. Although surgical time decreased in both these groups over the three-week period suggesting a learning curve, the robot-assisted group rotational and translational errors were lower in the robot-assisted group. The conclusion was that robot assistance helps reduce errors irrespective of experience [14]. A freehand sculpting semiactive robotic tool (NAVIO system, Blue Belt Technologies, Plymouth, MN) was reported to result in more accurate implant placement compared to other robot-assistive devices in synthetic and cadaveric femurs and tibia [15, 16].

Cobb et al. published their results of a randomised controlled trial of unicompartmental total knee arthroplasty performed in 27 patients (28 knees) either through conventional technique or with the assistance of the hands on robotic system, ACROBOT. All patients in the ACROBOT group had tibiofemoral alignment in the coronal plane within 2° of the planned position, while only 40% of the conventional group achieved this level of accuracy. There was a trend towards improvement in performance with increasing accuracy based on the Western Ontario and McMaster Universities Osteoarthritis Index and American Knee Society scores at six weeks and three months. There was no adverse effect but the surgical time was longer [17]. Wolf et al. reported on their experimental study performing patellofemoral arthroplasty using a mini bone attached robotic system (MBAR), an image-free system eliminating the need for external tracking. They proposed that this system improves accuracy and reduces operational time [18]. Turktas et al. demonstrated encouraging results in patellofemoral arthroplasty performed using robot assistance in patients at a mean follow-up of 15.9 months in 29 patients with 30 knees [19]. In prospectively followed 25 consecutive cases of total knee arthroplasty performed using an active robot, Bellemans et al. demonstrated excellent implant positioning and alignment within the 1° error of neutral alignment in all three planes in all cases. Despite this, they abandoned this procedure due to the excessive operating time required for the robotic implantation, the technical complexity of the system and the extremely high operational costs [20].

**Figure 2.**
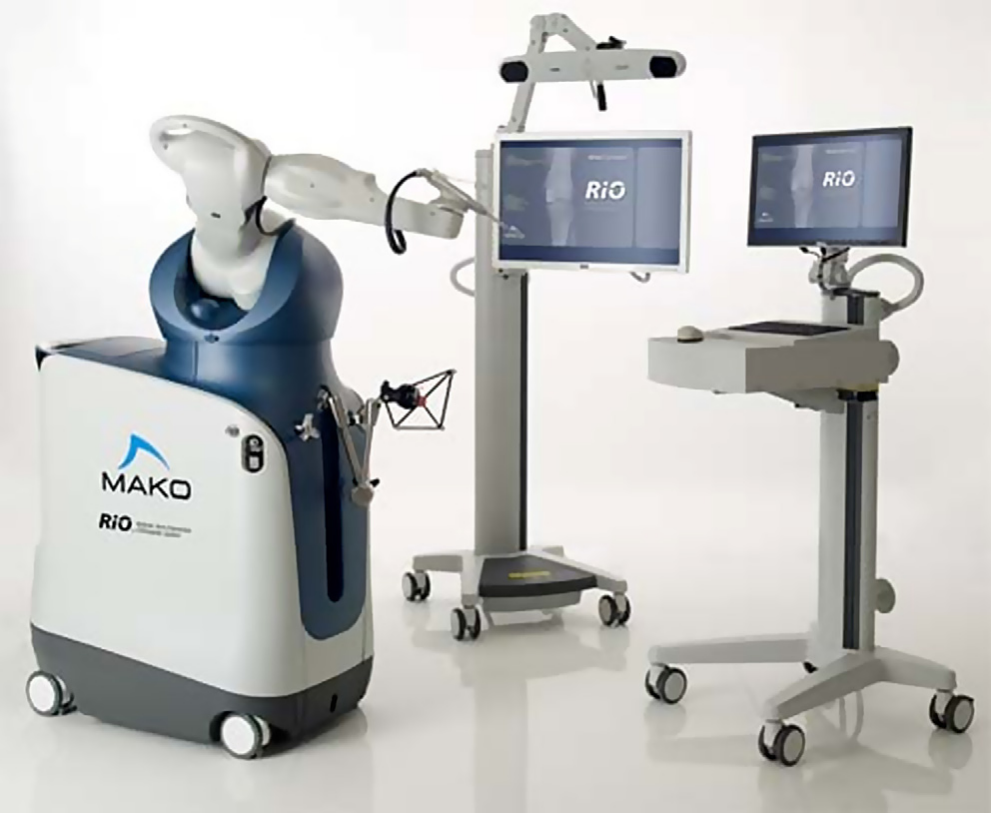
Mako Rio robot system.

The group led by Blythe in a prospective, randomised, single-blinded, controlled trial evaluating the accuracy of component positioning in unicompartmental knee arthroplasty compared robotic-assisted procedure using the MAKO Robotic Arm Interactive Orthopaedic (RIO) system (Figure 2) and conventional implantation techniques using the Oxford Phase-3 unicompartmental knee replacement. They assessed the accuracy of the axial, coronal and sagittal component positioning using a postoperative computed tomography scan at three months postoperatively. Of the 120 patients on whom they had data from the 139 patients included in the study 62 had robotic-assisted surgery and 58 underwent conventional implantation of the prosthesis. The proportion of patients with component implantation within 2° of the target position was significantly greater in the group who underwent robotic-assisted unicompartmental knee arthroplasty compared with the group who underwent conventional unicompartmental knee arthroscopy with regard to the femoral component in sagittal, coronal and axial position. The tibial component sagittal and axial position was also significantly better in the robotic-assisted group. They concluded that an improved accuracy of implant positioning in unicompartmental knee replacement can be obtained with the use of MAKO RIO (Stryker^®^) system compared to conventional techniques [21].

**Figure 3.**
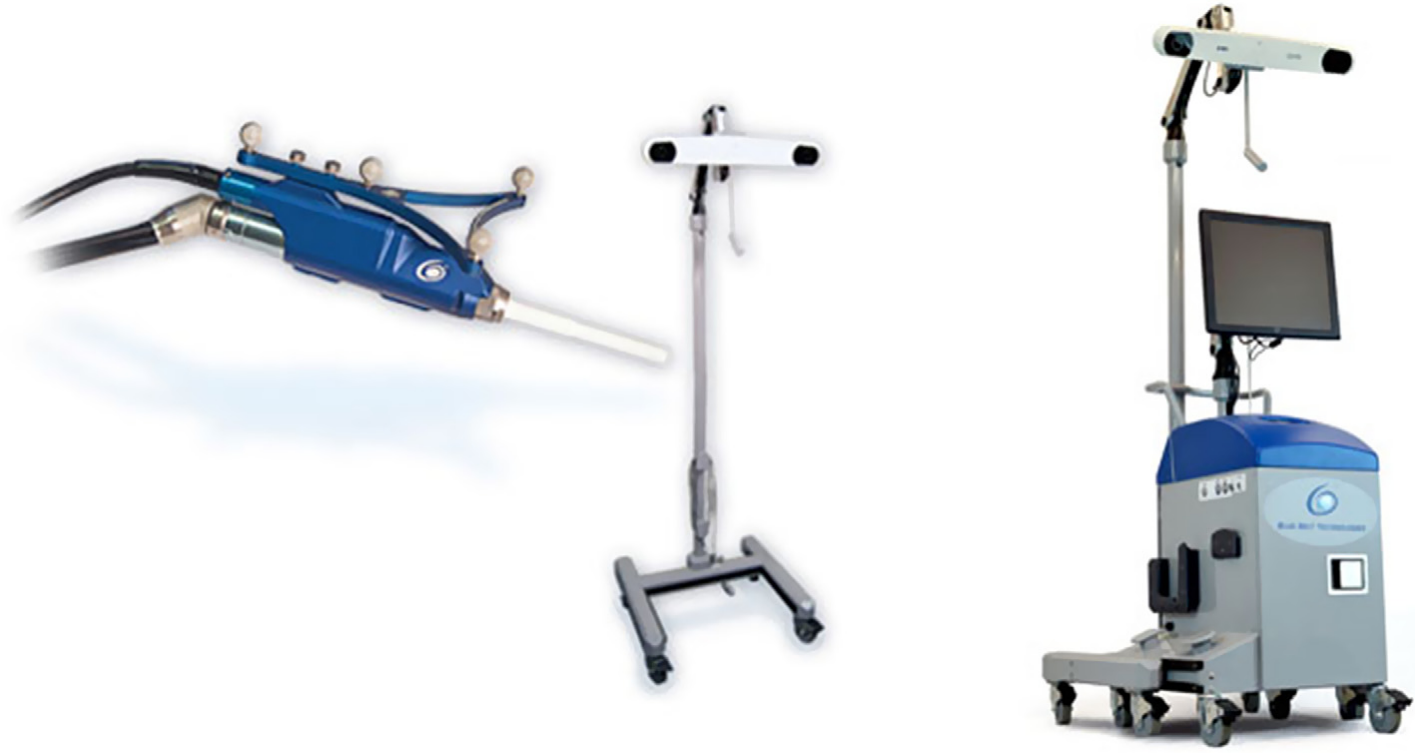
NAVIO robot with hand held precision sculpting tool.

Other clinical studies using the NAVIO handheld precision sculpting tool (Figure 3) for unicondylar knee arthroplasty reported improvement in radiographic alignment and Oxford knee scores postoperatively [22, 23]. The system has recently been acquired by Smith & Nephew^®^ and is currently being developed to expand into other subspecialties and total knee arthroplasty. The NAVIO system is an image-free system not reliant on preoperative CT scans but more on accurate intraoperative registration. The accuracy of implantation has been shown in cadaveric and synthetic models [15, 16] and further studies will be needed to show comparable clinical results. Both the MAKO and NAVIO systems offer a potential advance on previous navigation platforms as they allow accurate preoperative assessment and with the burring technique a potentially more accurate system to execute the plan than conventional cutting blocks with inherent saw deviations. The two platforms are a fusion of navigation and Robotics and it is important they have retained the ability for intraoperative checking of the cuts, which is an important factor in reducing compounding errors during knee surgery [5].

### Spine

The use of SpineAssist miniature robotic guidance system was described in 14 patients for the placement of pedicle screws. The SpineAssist performed successfully in 93% of the cases in which it was used, with 96% of the screws placed determined to be within 1 mm of their planned trajectory [24]. The technical and clinical challenges encountered in using this system were detailed by other authors [25]. In a retrospective multicentre study of robot-guided spine implant insertion with SpineAssist, the clinical acceptance of 3271 pedicle screws and guide wires inserted in 635 reported cases was assessed by intraoperative fluoroscopy and the placement accuracy of 646 pedicle screws inserted in 139 patients was measured using postoperative CT scans. Screw placements were found to be clinically acceptable in 98% of the cases when intraoperatively assessed by fluoroscopic images. Screws (98.3%) were within the safe zone on measuring with postoperative CT scans. Moreover, the SpineAssist system was safe and allowed percutaneous approach in 49% of cases [26]. Another prospective study using a new system, the Spine Bull’s-Eye Robot with the pedicle standard axis view (PSAV), demonstrated this system to be accurate and feasible in insertion of pedicle guide wires in the thoracic and lumbar spine in patients [27].

### Other applications

The use of robots has been described in cadavers in hip arthroscopy using the Da Vinci system [28], shoulder girdle and brachial plexus [29] and shoulder arthroscopy [30]. Robotic systems have also been described in experimental studies to guide intramedullary nailing and assist in distal locking of interlocking femoral nail [31, 32].

### Complications and challenges of robotic surgery

While robotic surgery helps improve the precision of implantation and bone sculpting there have been reported complications. These can be further classified as technical complications with the robot and surgical complications. Schulz et al. reported a technical complication rate of 9.3% with the ROBODOC system for hip arthroplasty [10]. This included the stoppage of bone milling needing reregistration during the procedure, fissuring of the femur, milling of a defect in the greater trochanter and damage to the acetabulum during milling. Moreover, they also reported 8.3% surgical complication rates, which included a 3.1% complication rate during Kirchner wire/pin insertion with damage to lateral cutaneous nerve of thigh, breakage of wire and knee effusion. Other surgical complications they reported were insertion of the cup at a suboptimal angle needing revision, femoral fissuring during repositioning and excess blood loss. Siebel and Käfer using the CASPAR system for hip arthroplasty reported a higher surgical time and an increased incidence of poor post op hip abductor function and incidence of Trendelenburg’s sign [33]. Similar technical and surgical complications have been reported with knee arthroplasty where procedures had to be abandoned due to failure of registration, robot workspace issues and damage to the patellar tendon [34, 35]. Errors in registration can have serious consequences due to the proximity of neurovascular structures around the hip and knee and have the potential to damage them in addition to the inaccuracies of bone milling. Other concerns that need addressing before universal adoption of robotic surgery are the increased operating time and increased costs associated with this technique making this procedure cost-effective only in high volume centres [36].

## Summary

Robots have been described for clinical use in hip arthroplasty, knee arthroplasty and spinal surgery. Experimental studies have explored their use in other orthopaedic surgery. In hip arthroplasty, robotic milling has been shown to result in improved rotatory stability and better bone implant contact in cementless arthroplasty. Clinical studies in hip arthroplasty further confirm better alignment but results in higher surgical time and higher risks of complications including recurrent dislocations while having no significant difference in long term outcome scores. Similar findings have been described in knee arthroplasty where there is an improvement in the alignment with robotic systems. Besides requiring increasing operating time there are technological challenges to this procedure. In spinal surgery, robots helped decrease exposure to fluoroscopy and results in more accurate placement of pedicle screws according to some studies. Surgical dissection is also facilitated, which enables minimally invasive procedures. Cost of the equipment remains a challenge. While robotic surgery has been demonstrated to be safe in many areas of orthopaedic surgery, their long term benefits and use in other areas including trauma are yet to be demonstrated. Due to the paucity of large clinical studies with long term outcomes, large-scale international collaborative studies with maintenances of registry data are recommended.

## Conflict of interest

The authors declare no conflict of interest in relation with this paper.
